# FDG-PET/CT in indeterminate thyroid nodules: cost-utility analysis alongside a randomised controlled trial

**DOI:** 10.1007/s00259-022-05794-w

**Published:** 2022-04-18

**Authors:** Elizabeth J. de Koster, Dennis Vriens, Maarten O. van Aken, Lioe-Ting Dijkhorst-Oei, Wim J. G. Oyen, Robin P. Peeters, Abbey Schepers, Lioe-Fee de Geus-Oei, Wilbert B. van den Hout, for the EfFECTS trial study group

**Affiliations:** 1 Department of Radiology and Nuclear Medicine, Radboud University Medical Centre, Nijmegen, the Netherlands; 2 Department of Radiology, Section of Nuclear Medicine, Leiden University Medical Center, Leiden, the Netherlands; 3 Department of Internal Medicine, Haga Hospital, The Hague, the Netherlands; 4 Department of Internal Medicine, Meander Medical Centre, Amersfoort, the Netherlands; 5 Department of Radiology and Nuclear Medicine, Rijnstate Hospital, Arnhem, the Netherlands; 6 Department of Biomedical Sciences and Humanitas Clinical and Research Centre, Department of Nuclear Medicine, Humanitas University, Milan, Italy; 7 Department of Internal Medicine, Erasmus University Medical Centre, Rotterdam, the Netherlands; 8 Department of Surgery, Leiden University Medical Center, Leiden, the Netherlands; 9 Biomedical Photonic Imaging Group, University of Twente, Enschede, the Netherlands; 10 Department of Biomedical Data Sciences, Leiden University Medical Center, Leiden, the Netherlands

**Keywords:** [^18^F]FDG-PET/CT, Indeterminate thyroid nodule, Thyroid carcinoma, Thyroid surgery, Cost-effectiveness, Costs, Health-related quality of life, QALY

## Abstract

**Purpose:**

To evaluate cost-effectiveness of an [^18^F]FDG-PET/CT-driven diagnostic workup as compared to diagnostic surgery, for thyroid nodules with Bethesda III/IV cytology. [^18^F]FDG-PET/CT avoids 40% of futile diagnostic surgeries for benign Bethesda III/IV nodules.

**Methods:**

Lifelong societal costs and quality-adjusted life years (QALYs) were assessed for 132 patients participating in a randomised controlled multicentre trial comparing [^18^F]FDG-PET/CT to diagnostic surgery. The observed 1-year trial results were extrapolated using a Markov model. The probability of cost-effectiveness was estimated using cost-effectiveness acceptability curves, taking uncertainty about sampling, imputation, and parameters into account.

**Results:**

The observed 1-year cost difference of [^18^F]FDG-PET/CT as compared to diagnostic surgery was − €1000 (95% CI: − €2100 to €0) for thyroid nodule–related care (p = 0.06). From the broader societal perspective, the 1-year difference in total societal costs was − €4500 (− €9200 to €150) (p = 0.06). Over the modelled lifelong period, the cost difference was − €9900 (− €23,100 to €3200) (p = 0.14). The difference in QALYs was 0.019 (− 0.045 to 0.083) at 1 year (p = 0.57) and 0.402 (− 0.581 to 1.385) over the lifelong period (p = 0.42). For a willingness to pay of €50,000 per QALY, an [^18^F]FDG-PET/CT-driven work-up was the cost-effective strategy with 84% certainty.

**Conclusion:**

Following the observed reduction in diagnostic surgery, an [^18^F]FDG-PET/CT-driven diagnostic workup reduced the 1-year thyroid nodule–related and societal costs while sustaining quality of life. It is very likely cost-effective as compared to diagnostic surgery for Bethesda III/IV nodules.

**Trial registration number:** This trial is registered with ClinicalTrials.gov: NCT02208544 (5 August 2014), https://clinicaltrials.gov/ct2/show/NCT02208544.

**Supplementary Information:**

The online version contains supplementary material available at 10.1007/s00259-022-05794-w.

## Introduction

Thyroid malignancy is detected in approximately one in four cytological indeterminate thyroid nodules, including cytology with atypia of undetermined significance or follicular lesions of undetermined significance (Bethesda III, AUS/FLUS) and cytology (suspicious for a) follicular neoplasm (Bethesda IV, FN/SFN) or (suspicious for a) Hürthle cell neoplasm (Bethesda IV, HCN/SHCN) [1]. Current guidelines recommend repeat fine needle aspiration cytology (FNAC) in Bethesda III nodules, and consideration of clinical features, ultrasound characteristics and patient preference in both Bethesda III and IV nodules, before deciding to proceed with either active surveillance or diagnostic surgery [1–4]. In the Netherlands, from 2017 to 2019, approximately 1300 Bethesda III and 650 Bethesda IV cytology results were reported per year. Many of these patients underwent diagnostic surgery [5]. Better preoperative differentiation could avoid futile diagnostic surgeries for benign nodules of indeterminate cytology, including the associated costs, risks of surgical complications, lifelong thyroid hormone substitution in patients with subsequent hypothyroidism, and possible negative influence on the patients’ health-related quality of life (HRQoL) [6–8]. However, none of the plethora of available additional diagnostics are currently part of the standard diagnostic workup following national or international guidelines [4, 9–12].

Our recent randomised controlled multicentre trial confirmed the results of our previous meta-analysis and demonstrated that implementation of 2-[^18^F]fluoro-2-deoxy-D-glucose positron emission tomography/computed tomography ([^18^F]FDG-PET/CT) in the preoperative workup accurately ruled out malignancy and prevented 40% of the futile diagnostic surgeries for benign nodules [7]. If the application of [^18^F]FDG-PET/CT is limited to nodules with non-Hürthle cell cytology (AUS/FLUS and FN/SFN), a 48% reduction can be established, optimizing therapeutic yield and limiting the unbeneficial use of valuable resources [13].

Prior to the implementation of any new test or procedure, it is crucial to evaluate cost-utility. We previously reported a model-based cost-utility analysis of [^18^F]FDG-PET/CT in a European setting, which demonstrated that [^18^F]FDG-PET/CT could be cost-effective as compared to management with diagnostic surgery or molecular testing over a 5-year period [7, 14]. To the best of our knowledge, no cost-utility analysis was performed alongside a clinical trial to date, even though such a design would offer a high level of evidence and a most accurate reflection of real-world clinical practice. Here, we present the results of the cost-utility analysis derived from our randomised controlled multicentre trial. In this analysis, we compared the lifelong societal costs and quality adjusted life years (QALYs) of an [^18^F]FDG-PET/CT-driven workup to the costs and QALYs of diagnostic surgery in patients with indeterminate thyroid nodules. The observed and prospectively collected 1-year trial outcomes were extrapolated using a Markov model.

## Material and methods

### Trial design, patients and treatment

**Fig. 1:**
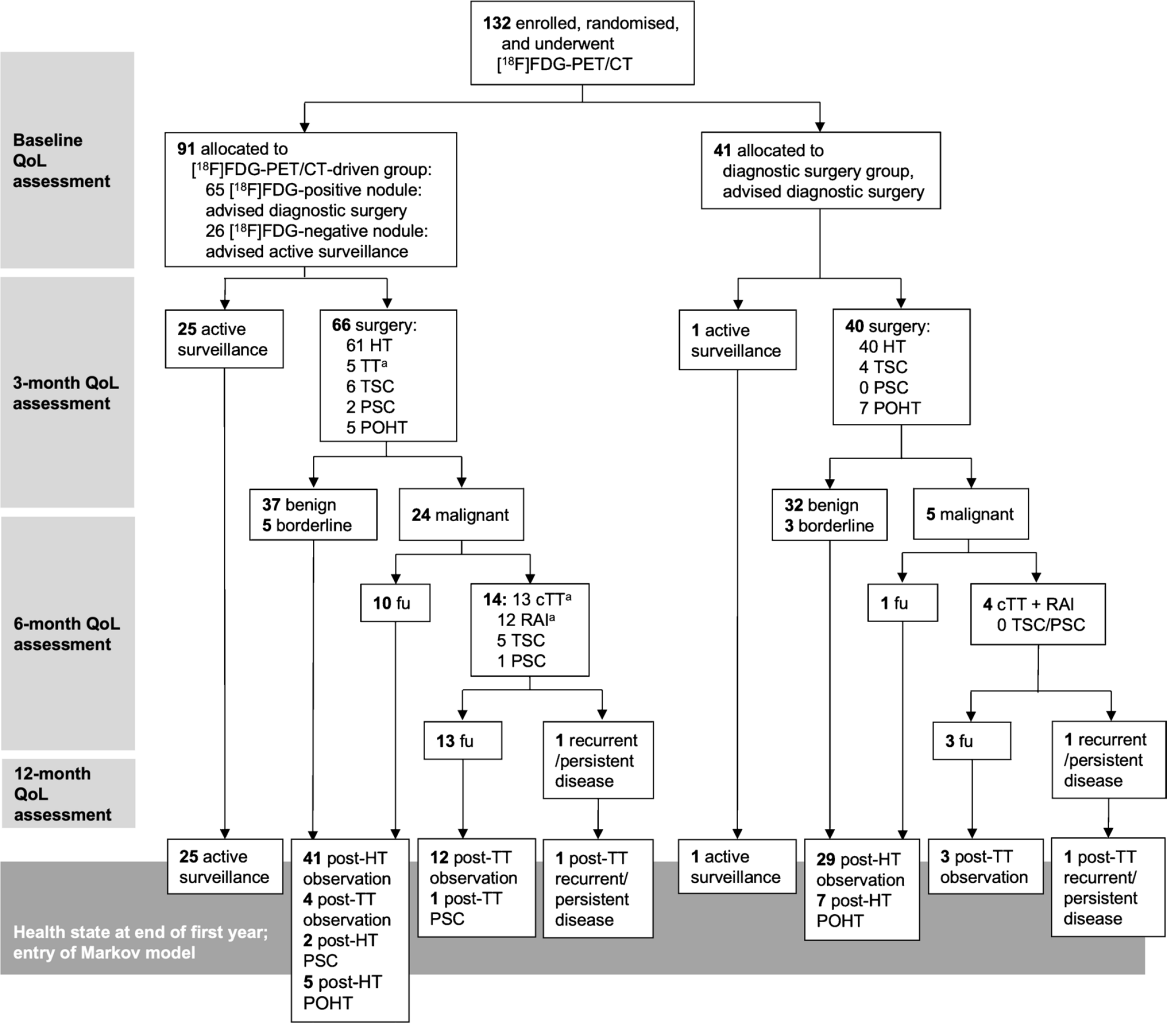
Flowchart of the first year, visualizing the study procedures, observed treatment and treatment outcomes, and health state at the end of the first year of all patients who participated in the *EfFECTS* trial. cTT, completing total thyroidectomy. Fu, follow-up. HT, hemithyroidectomy (including isthmus resection (n = 3) and hemithyroidectomy plus nodulectomy (n = 2). POHT, postoperative levothyroxine-dependent hypothyroidism after partial thyroidectomy procedure. PSC, permanent surgical complication, including recurrent nerve paralysis and permanent hypoparathyroidism. RAI, radioiodine ablative therapy. TSC, transient surgical complication, including haematoma with re-exploration surgery, wound infection, seroma, and transient hypoparathyroidism. TT, total thyroidectomy. ^a^: One patient underwent RAI after initial, uncomplicated TT for malignancy; two patients underwent cTT for malignancy but no RAI

The *Efficacy of [*^*18*^*F]FDG PET in Evaluation of Cytological indeterminate Thyroid nodules prior to Surgery (EfFECTS)* trial was a prospective, triple-blinded, randomised controlled multicentre trial performed in 15 hospitals in the Netherlands (ClinicalTrials.gov: NCT02208544). The trial, including the current study, was approved by the Medical Research Ethics Committee on Research Involving Human Subjects region Arnhem-Nijmegen, Nijmegen, the Netherlands. Written informed consent was obtained from each of the participants prior to any study activity. Comprehensive descriptions regarding patient eligibility, selection, randomisation, blinding, [^18^F]FDG-PET/CT procedures, and sample size calculation are reported in our previous work [13]. In summary, patients with a Bethesda III or IV thyroid nodule (confirmed on central review; Bethesda III on repeat FNAC) and scheduled diagnostic surgery were eligible for inclusion (Table 1). There was one index nodule per patient. Patients were randomly assigned to an [^18^F]FDG-PET/CT-driven group or diagnostic surgery group in a 2:1 ratio (Fig. 1). Randomisation was stratified for patient sex, age, thyroid nodule size, Bethesda classification (III or IV), and inclusion site. A partial-body [^18^F]FDG-PET/CT of the neck was acquired in all patients, and centrally assessed by two experienced nuclear medicine physicians for any focal [^18^F]FDG-uptake in the thyroid that was visually higher than the background uptake of the surrounding thyroid tissue and that corresponded to the index nodule in size and location. Patient allocation and the result of the [^18^F]FDG-PET/CT scan were not disclosed to the patient nor his/her local physician. Subsequently, the recommended patient management in the [^18^F]FDG-PET/CT-driven group was based on the result of the scan. When the index nodule was [^18^F]FDG-positive, patients were advised to proceed to the scheduled diagnostic surgery. When the index nodule was [^18^F]FDG-negative, active surveillance was recommended, with at least a follow-up ultrasound after one year. Any additional follow-up visits during the trial were permitted at the discretion of the local physician. In the diagnostic surgery group, all patients were advised to proceed to the scheduled surgery, in accordance with current (inter)national guidelines [4, 12]. In all patients in both groups, postoperative management was based on the local histopathological diagnosis and adhered to the Dutch national guidelines [12]. The current study adhered to this *local* histopathological diagnosis as a reference standard, as this diagnosis likely best reflects the patient’s illness perception and estimated costs. Consequently, minor differences exist between the current study and the trial’s main report, for which all histopathology was centrally reviewed [13]. Index nodules diagnosed as non-invasive follicular thyroid neoplasm with papillary-like nuclear features (NIFTP) or follicular tumour of uncertain malignant potential (FT-UMP) are considered borderline tumours: they were postoperatively treated as benign nodules, but diagnostic surgery for these potentially premalignant nodules is considered justified [15, 16]. The study-related follow-up for all patients was 1 year.

**Table 1:** Baseline characteristics of the patients enrolled in the trial

	FDG-PET/CT driven management	diagnostic surgery	
	*n* = 91	*n* = 41	*p*
Female sex — n (%)	73 (80%)	34 (83%)	0.71^a^
Age at baseline (years) (mean ± SD)	54.3 ± 14.6	54.5 ± 11.6	0.95^b^
General medical history — *n* (%)	81 (89%)	33 (81%)	0.19^a^
Cardiovascular disease (including stroke)	29 (32%)	12 (29%)	0.77^a^
Non-thyroid solid malignancy	8 (9%)	5 (12%)	0.54^c^
Haematological disease or malignancy	8 (9%)	4 (10%)	1^c^
Neurological disease (excluding stroke)	19 (21%)	9 (22%)	0.89^a^
Otolaryngological disease	17 (19%)	9 (22%)	0.66^a^
Lung disease	13 (14%)	7 (17%)	0.68^a^
Gastro-intestinal disease	26 (29%)	9 (22%)	0.43^a^
Urological or gynaecological disease	29 (32%)	16 (39%)	0.42^a^
Endocrine disease (excluding thyroid)	17 (19%)	6 (15%)	0.57 ^a^
Musculoskeletal disorder	33 (36%)	17 (41%)	0.57 ^a^
Psychiatric disorder	5 (6%)	2 (5%)	1^c^
[^18^F]FDG-PET/CT — *n* (%)			
[^18^F]FDG-positive nodule	65 (71%)	26 (63%)^d^	0.36^a^
Incidental findings on [^18^F]FDG-PET/CT	25 (27%)	16 (39%)^d^	0.23^a^
[^18^F]FDG-positive incidentaloma	10 (11%)	9 (22%)^d^	0.10^a^
Diagnosis — *n* (%)^e^			
Malignant	24 (26%)	5 (12%)	0.07^a,e^
Borderline	5 (5%)	3 (7%)	
Benign on histopathology	37 (41%)	32 (78%)	
Benign on ultrasound follow-up	25 (27%)	1 (2%)	
Treatment — *n* (%)			
Diagnostic surgery	66 (73%)	40 (98%)	**0.001**^a^
Watchful waiting	25 (27%)	1 (2%)	
Completion thyroidectomy	13 (14%)	4 (10%)	0.58^c^
RAI	12 (13%)	4 (10%)	0.78^c^
Productivity (n = 121) — *n* (%)^f^			
Full-time job	15 (17%)	10 (29%)	0.39^a^
Part-time job	41 (48%)	14 (40%)	
Unemployed	30 (35%)	11 (31%)	
Average contractual work hours (hours/week) — median (IQR)	20 (0–30)	24 (0–36)	0.29^d^

Further comprehensive baseline characteristics, including cytological classification and [^18^F]FDG-PET/CT parameters, are presented in our previous work [13]. *IQR*, interquartile range. *RAI*, radioiodine ablative therapy. ^a^: Pearson chi square. ^b^: independent samples t-test. ^c^: Fisher’s exact test. ^d^: In the current study, costs related to the [^18^F]FDG-PET/CT are not taken into account for the diagnostic surgery group. [^18^F]FDG-PET/CT data for the diagnostic surgery group are presented here solely for comparison of baseline characteristics. ^e^: data presented here are local histopathological diagnoses, and include minor discordances as compared to the centrally reviewed histopathology diagnoses presented in the *EfFECTS* trial’s main paper [13]. ^f^: 121 patients completed the baseline iPCQ questionnaire. ^g^: Mann–Whitney U test

### First year costs and utilities

Real-world volumes of thyroid nodule-related health care consumption for 1 year, counted from the date of the [^18^F]FDG-PET/CT scan (defined as baseline), were extracted from individual medical records for each patient. The extracted data included all thyroid surgery and associated days of hospitalization, additional procedures and days of hospitalization following surgical complications, outpatient clinic visits and diagnostics that were related to the diagnosis and treatment of the indeterminate thyroid nodule, additional diagnostic procedures and consultations with other physicians related to [^18^F]FDG-PET/CT incidental findings, and use of thyroid-related medication. Volumes concerning non-thyroid-related health care consumption, productivity losses and HRQoL during the first year were patient-reported at baseline, 3, 6 and 12 months, using the iMTA Medical Consumption Questionnaire (iMCQ), the iMTA Productivity Costs Questionnaire (iPCQ) and the EuroQol 5-dimension 5-level (EQ-5D-5L) questionnaire, respectively (Fig. 1) [17–19]. Questions on health care and productivity covered a fixed recall period by design of each questionnaire, varying from one to 3 months; intermediate periods were individually interpolated from the closest available questionnaire. Utilities were calculated from the EQ-5D-5L domain scores using the Dutch tariff [20]. These utilities represent the valuation of quality of life on a scale from 0 (worst possible health, similar to death) to 1 (perfect health). Quality adjusted life years (QALYs) for the first year were estimated as the area under the utility curve [20, 21].

The estimated cost of one partial-body [^18^F]FDG-PET/CT scan was €754 [22, 23]. Other health care costs were valued using reference prices or the 2019 reimbursement rates of the Dutch System of Diagnosis-Treatment Combinations, where appropriate and available [23]. Costs for complications of thyroid surgery (i.e., prolonged hospitalization, re-admission, and/or additional surgical procedures) were estimated using complication rates reported in literature and procedural Dutch reimbursement rates [22]. Costs of productivity losses were valued using the friction cost method and reference prices for productivity [23]. Travel expenses were included at €0.19 per kilometre [23]. We estimated all costs from a Dutch societal perspective in Euro. All prices were indexed to 1 December 2019 using the Dutch consumer price index [24].

The total societal costs per patient were estimated as the sum of medical costs for all thyroid nodule-related and other health care consumption, patient costs (i.e., travel expenses and informal care), and costs from productivity losses. All costs related to the [^18^F]FDG-PET/CT, including procedure costs, costs for additional healthcare consumption for incidental [^18^F]FDG-PET/CT findings, pertinent travel expenses, and other reported patient costs were only taken into account for the patients in the [^18^F]FDG-PET/CT-driven group.

Multiple imputation was applied to account for possibly selectively missing questionnaire data, using age, sex, allocation, EQ-5D-5L utility scores and time-dependent variables for thyroid surgery and benign or malignant histopathological diagnosis as predictor variables. One hundred imputed datasets were created for the 1-year data.

### Modelled lifelong costs and utilities

To estimate lifelong costs and utilities, a Markov model with 12 health states and a 1-year cycle length was constructed using Stata (version 14.2. StataCorp, College Station, TX, USA).

#### Model structure

**Fig. 2:**
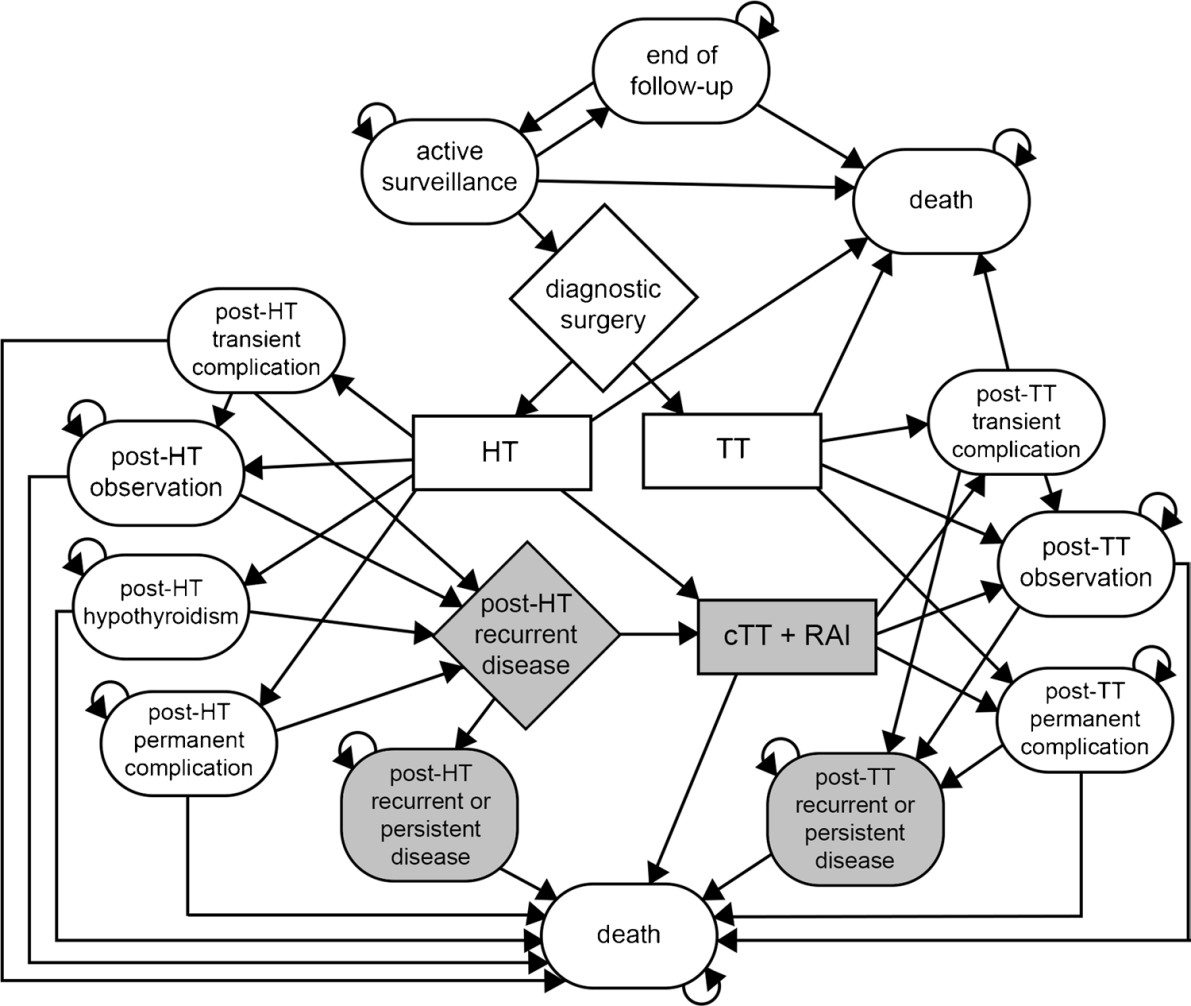
Markov tree visualizing the health states (ovals), possible transitions between health states after each 1-year cycle length (arrows), treatments (boxes), and decisions (diamonds) that patients may encounter in the Markov model. Patients enter the model in their actual health state at the end of the first year. Grey-shaded fields and corresponding transitions only apply to patients with malignancy; all white fields and corresponding transitions apply to patients with either benign or malignant lesions, although different (transition) probabilities, costs, and utilities may apply as presented in Tables 2 and 3. cTT, completing total thyroidectomy. HT, hemithyroidectomy. RAI, radioiodine ablative therapy. TT, total thyroidectomy

The model represented health states that may occur from the second year onwards for either an [^18^F]FDG-PET/CT-driven workup or diagnostic surgery (Fig. 2). These health states included active surveillance (i.e., follow-up of the thyroid nodule with yearly ultrasound), end of follow-up (i.e., patients discharged from active surveillance without thyroid surgery), observation after thyroid surgery (i.e., hemithyroidectomy [HT], total thyroidectomy [TT], completion TT [cTT], and/or radioactive iodine [RAI] ablation), medication-dependent hypothyroidism following HT, permanent complications due to HT or (c)TT, recurrent (including persistent) malignant disease after HT or (c)TT and/or RAI, or death. Health states following HT or TT may apply to patients with either benign or malignant disease. The “cTT + RAI” procedure and recurrent disease states (grey-shaded shapes in Fig. 2) only apply to patients with malignant disease.

#### Model parameters

Values for the (time-dependent) probabilities in the Markov model were collected from a comprehensive Medline literature search, from Statistics Netherlands, and/or from the *EfFECTS* trial and adhered to the Dutch national guidelines (Table 2) [4, 12, 13, 25]. Parameters for which no information was found or that varied highly among literature were estimated by a local expert panel, including an endocrinologist, a nuclear medicine physician, and a health economist. For patients undergoing active surveillance for an [^18^F]FDG-negative nodule, a mean follow-up of 3 years was assumed.

**Table 2:** Transition probabilities for the Markov Model, including uncertainty

	Base-case value	Source	Uncertainty^a^
**Discount rates**			
Yearly discount rates for costs	0.040	[14, 23]	Fixed
Yearly discount rates for utilities	0.015	[14, 23]	Fixed
**Follow-up of [**^**18**^**F]FDG-negative nodules**			
Yearly probability that active surveillance of [^18^F]FDG-negative nodule ends	0.33 (based on mean 3-year f/u)	[36, 37, 40, 41], expert opinion	0.2–1.0
Yearly probability to re-enter active surveillance for [^18^F]FDG-negative nodule	0.01	Expert opinion	− 50% to + 100%
Yearly probability of surgery for benign lesion after continued surveillance for [^18^F]FDG-negative nodule	0.02	[13, 36–38], *EfFECTS*, expert opinion	− 50% to + 100%
Probability of (surgery for) a missed malignancy after initial surveillance for [^18^F]FDG-negative nodule	0.049 (1-NPV)	[7, 13, 42], *EfFECTS*	0.0–0.1
Maximum timespan for missed malignancy to be detected	5 years	Expert opinion	2–15 years
Fraction HT of all surgery	0.95	[14], expert opinion	0.90–0.98
Fraction of cTT following HT if malignant	0.607	*EfFECTS*	0.5–0.7
**Recurrence of malignancy**			
Yearly probability of recurrent disease following HT	Year 1–5: 0.015 Year 6–10: 0.010 Year 11 onwards: 0.005	[43–51]	0.0075–0.03 0.005–0.02 0.0025–0.01
Probability of (c)TT following recurrence after HT for malignancy	0.917	[14]	SD = 0.013
Yearly probability of recurrent or persistent malignant disease following (c)TT	Year 1–2: 0.070 Year 3–5: 0.040 Year 6–10: 0.010 Year 11 onwards: 0.005	[4, 14, 43, 47–58], *EfFECTS*	− 50% to + 100%
**Mortality**			
Yearly probability of death of any cause (not thyroid cancer related)	Life tables	[25]	Fixed
Yearly probability of death due to thyroid cancer	Year 1–10: 0.005 Year 11–20: 0.003 Year 21 onwards: 0.002	[14, 46–48, 50–52, 54, 59–61]	− 50% to + 100%
Perioperative mortality HT/(c)TT	0.0011	[8, 14, 62]	− 50% to + 100%
**Complications of thyroid surgery**			
Transient complication due to HT	0.0977	[8, 13, 14, 26, 29, 62–68]	− 50% to + 100%
Permanent complication due to HT (excluding hypothyroidism)	0.0056	[8, 13, 14, 26, 29, 62, 63, 67, 68]	− 50% to + 100%
Medication-dependent hypothyroidism due to HT	0.22	[13, 26, 27, 29, 30, 69]	SD = 0.020
Transient complication due to (c)TT	0.185	[8, 14, 26, 62–68, 70, 71], *EfFECTS*	− 50% to + 100%
Permanent complication due to (c)TT	0.046	[8, 14, 26, 62, 63, 67, 68, 70], *EfFECTS*	− 50% to + 100%

^a^ Ranges are for triangular parameter distributions (with mode equal to the base-case value), except when the parameter is fixed or has a normal distribution (as indicated by the SD). *DTC*, differentiated thyroid carcinoma. *EfFECTS*, observed data from the first year of the *EfFECTS* trial were included as a source. HT, hemithyroidectomy. (c)TT, (completing) total thyroidectomy. NPV, negative predictive value. SD, standard deviation

The negative predictive value (NPV) of [^18^F]FDG-PET/CT was 95.1% in the *EfFECTS* trial [13]. To prevent overestimation of the accuracy of an [^18^F]FDG-PET/CT-driven workup, we used this NPV to assume a 0.049 (= 1–0.951) probability of missed malignancies in unoperated patients (i.e., a false-negative [^18^F]FDG-PET/CT), even though none were reported in the *EfFECTS* trial and its extended follow-up [13]. We assumed that any missed malignancies would be detected within the first 5 years of follow-up, and could occur among patients residing in the “active surveillance” or “end of follow-up” state (Table 2).

#### Cost parameters

Costs for thyroid-related procedures and costs for each cycle in a particular health state were derived from reference prices, 2019 reimbursement rates, and previous cost-utility studies, where appropriate and available, and adhered to the national guidelines (Table 3) [14, 22, 23, 26–30]. For the “active surveillance” state, we assumed one yearly visit to the endocrinologist and an ultrasound of the neck every 12–24 months.

**Table 3:** Costs and utilities for the Markov Model, including uncertainty

	Costs, Base-case value	Source	Uncertainty^a^	Disutility, Base-case value^b^	Source	Uncertainty^a^
**Procedures**						
FDG-PET/CT	€754	[22]	n.a			
Hemithyroidectomy	€4315	[22]	± 25%			
Total / completion thyroidectomy	€6115	[22]	± 25%			
Radioiodine ablation	€5765	[22]	± 25%			
**Health states, yearly costs**						
Active surveillance after negative [^18^F]FDG-PET/CT^c^	€236	[4, 14, 22, 26, 27, 29, 38, 41, 72], expert opinion	± 25%	0.02	[14, 26, 28]	0.00–0.05
End of follow-up	€0	Expert opinion	€0	0.01	Expert opinion	0.00–0.04
Observation after HT for benign nodule						
1^st^ year	€277	[14, 22, 26, 28, 30, 73]	± 25%	0.01	[14, 26, 28, 74]	0.00–0.04
2^nd^ year onwards	€0	[14, 28, 73]	± 25%	0.01	[14, 26, 28, 74]	0.00–0.04
Observation after HT for malignancy						
1^st^ year	€529	[12, 22, 28, 73]	± 25%	0.03	[14, 26, 28, 74], expert opinion	0.01–0.06
2^nd^–5^th^ year	€252	[12, 22, 28, 73]	± 25%	0.02	[14, 26, 28, 74], expert opinion	0.00–0.05
6^th^ year onwards	€0	[12, 22, 28, 73]	± 25%	0.01	[74]	0.00–0.04
Transient complication due to HT	€1272	[14, 22, 26–29, 73, 75], *EfFECTS*	± 25%	0.06	[14, 26, 28]	0.02–0.11
Permanent complication due to HT						
1^st^ year	€5338	[14, 26–30, 72, 73, 75], *EfFECTS*	± 25%	0.30	[14]	0.21–0.39
2^nd^ year onwards	€825	[14, 26, 28, 72, 73, 75]	± 25%	0.30	[14]	0.21–0.39
Hypothyroidism due to HT						
1^st^ year	€566	[22, 26, 30, 72, 75], *EfFECTS*	± 25%	0.03	[14, 26, 28, 30]	0.01–0.06
2^nd^ year onwards	€283	[22, 26, 30, 72, 75]	± 25%	0.02	[14, 26, 28, 30]	0.00–0.05
Recurrence of malignancy after HT	€1756	[14, 28]	± 25%	0.40	[14]	0.31–0.50
Observation after TT for benign nodule						
1^st^ year	€843	[14, 22, 26, 28, 30, 72, 73, 75]	± 25%	0.06	[14, 26, 28, 30]	0.02–0.11
2^nd^ year onwards	€283	[14, 22, 26, 28, 30, 72, 73, 75]	± 25%	0.02	[14, 26, 28, 30]	0.00–0.05
Observation after (c)TT for malignancy						
1^st^ year	€1949	[12, 14, 22, 28]	± 25%	0.07	[14, 26, 28, 74]	0.03–0.12
2^nd^–15^th^ year	€753	[12, 14, 22, 28]	± 25%	0.04	[14, 26, 28, 74]	0.02–0.07
16^th^ year onwards	€0	[12]	± 25%	0.02	Expert opinion	0.00–0.05
Transient complication due to (c)TT	€1106	[14, 22, 26–29, 73, 75]	± 25%	0.06	[14, 26, 28]	0.02–0.11
Permanent complication due to (c)TT						
1^st^ year	€3462	[14, 26–30, 72, 73, 75]	± 25%	0.35	[14, 26, 28]	0.26–0.45
2^nd^ year onwards	€722	[14, 26, 28, 72, 73, 75]	± 25%	0.35	[14, 26, 28]	0.26–0.45
Recurrence after (c)TT	€1452	[14, 28]	± 25%	0.40	[14, 28]	0.31–0.50
Death	€0	Convention		0	Convention	Fixed
**Other health-care related costs, yearly costs**						
Other health care consumption^d^	€2511	*EfFECTS*				
Travel expenses for thyroid-related health care^e^	€12–€105	*EfFECTS*	± 25%			
Travel expenses for other health care consumption^d^	€ 99	*EfFECTS*				
Informal care^d^	€ 604	*EfFECTS*				
**Productivity losses, yearly**						
Productivity losses due to HT						
HT for benign nodule	€3065	*EfFECTS*	€ 620 (SD)			
HT for malignant nodule	€3925	*EfFECTS*	€ 1,238 (SD)			
Productivity losses due to total / completion thyroidectomy	€4686	[76–79], *EfFECTS*	€ 1,028 (SD)			
Productivity losses due to RAI	€1188	[79–83], expert opinion	€ 292 (SD)			
Yearly productivity losses for recurrent/ progressive malignant disease	€2493	[76–78, 80, 84–86]	± 25%			
Yearly other paid productivity losses^d^	€ 2267	*EfFECTS*				
Yearly unpaid productivity losses^d^	€ 1153	*EfFECTS*				

^a^ Ranges are for triangular parameter distributions (with mode equal to the base-case value). ^b^ Subtracted from age and sex dependent utilities [20]. ^c^ Active surveillance was defined as a yearly visit to the endocrinologist and an ultrasound of the neck every 12–24 months. ^d^ Linear regression analysis was performed using the first-year trial data to establish estimates for this variable, including sex, age, and estimated QALYs as predictors. Reported values in this table are parameter means; more detailed regression analysis data, including uncertainty, are provided in Supplementary Table 1. ^e^ Costs are dependent on the model health state, see Supplementary Table 2. *HT*, hemithyroidectomy. (c)TT, (completing) total thyroidectomy. *EfFECTS*, observed data from the first year of the *EfFECTS* trial were included as a source. *RAI*, radioiodine ablative therapy; *SD*, standard deviation

Productivity losses for thyroid-related procedures were inferred from the reported iPCQ data over the first year of the *EfFECTS* trial or from literature, where appropriate. Yearly costs for other non-thyroid-related health care consumption, informal care, and other productivity losses were estimated from the reported first-year cost-questionnaire data in our study, using restricted linear regression analysis with age, sex, and QALYs as predictors (restricting coefficients to predict non-negative costs) (Supplementary Table 1). Travel expenses were estimated from the number of hospital visits for each procedure or health state, and the patient-reported travel distance.

#### Utility parameters

Utilities were calculated, starting from age and sex-dependent general utilities [20], by subtracting disutilities for specific health states (Table 3). These disutilities were derived from literature or elicited from the previously mentioned expert panel based on a time-trade-off weighting. QALYs were calculated by the discounted sum of utilities over the lifelong evaluation period.

#### Other parameters

A 4% and 1.5% discount rate were applied to all future costs and utilities, respectively [23]. In addition to the base-case values, distributions were specified to account for the uncertainty in the parameters. These were either triangular parameter distributions (on a specified range, with mode equal to the base-case value) or normal distribution (with specified SD and mean equal to the base-case value).

#### Lifelong extrapolation

With each of the 100 imputed 1-year datasets, 10 sets of model parameter values were drawn at random from the specified parameter distributions. Then, for each of the 1000 parameter sets and starting from each patient’s health state at the end of the first year, the Markov model was used to simulate 1000 extrapolated patient histories. For each parameter set, the average over the extrapolated costs and QALYs was added to the 1-year costs and QALYs, as an estimate of the patients’ expected lifelong outcomes.

### Statistical analysis

Baseline characteristics were compared between the allocated groups using Pearson’s chi-squared or Fisher’s exact tests for categorical data, and independent samples t-tests or Mann–Whitney U tests for continuous data, where appropriate. Univariate comparisons of the 1-year costs and QALYs were performed using independent unequal-variances t-tests, aggregating the 100 multiple imputation sets using Rubin’s rules (accounting for sampling and imputation uncertainty). Similarly, lifelong costs and QALYs were compared by aggregating the 1000 parameter sets using Rubin’s rules (accounting for sampling, imputation and parameter uncertainty) [31]. Unadjusted (univariate) results are presented in the Supplementary data.

In the analyses presented here, we adjusted for the trial’s stratifying variables using a generalized linear model with robust estimator for observed heteroscedastic data [13, 32, 33].

Minor imbalances in baseline characteristics and malignancy rates were observed across the allocated groups despite stratified randomisation (Table 1). To avoid an impact of these imbalances on costs and utilities over the lifelong period, we also adjusted for these covariates: the *local* benign/borderline or malignant histopathological diagnosis, EQ-5D-5L utility score at baseline, medical history (binary, represented by the periodic use of non-thyroid medication), and productivity at baseline (represented by the patient-reported contractual work hours per week. Unadjusted results are presented in Supplementary Tables 3, 4, 5 and Supplementary Fig. 1. Results are presented as means and their 95% confidence intervals (CI), mean difference and 95% CI, and *p* values, where appropriate. All analyses adhered to the intention-to-treat principle. A *p* value ≤ 0.05 is considered statistically significant. Data analysis was performed using SPSS Statistics version 26 (IBM Corp., Armonk, NY, USA).

### Cost-utility analysis

Cost-effectiveness acceptability curves (CEACs) were used to graph the probability that an [^18^F]FDG-PET/CT-driven workup is cost effective compared to diagnostic surgery, as a function of willingness to pay (WTP) for a QALY. In the Netherlands, a willingness-to-pay threshold of €50,000 per QALY is recommended by the Dutch Council for Public Health and Health Care for conditions with an intermediate disease burden [34]. The probability of cost-effectiveness was calculated as the one-sided *p* value for the difference in net benefit (net benefit = WTP × QALYs − costs). The statistical analysis of the net benefit was identical to the analysis for costs and QALYs separately.

To explore the impact of individual parameters in the Markov model, univariate sensitivity analyses were performed and presented in a tornado diagram. Individual parameters were set at extreme values (Table 4), while keeping the other parameters at their base-case value and for each trial patient simulating 10,000 extrapolated patient histories beyond 1 year.

**Table 4:** Probabilities, costs, and utilities for univariate sensitivity analyses

	Range	References
**Probabilities**		
Yearly probability that active surveillance of [^18^F]FDG-negative nodule ends	0.05–1.00	[36, 37, 40, 41], expert opinion
Yearly probability of surgery for benign nodule after continued surveillance for [^18^F]FDG-negative nodule	0.001–0.10	[14, 27, 36–38], *EfFECTS*
Yearly probability of (surgery for) a missed malignancy after initial surveillance for [^18^F]FDG-negative nodule	0.00–0.05	[7, 26, 29, 42], *EfFECTS*
Any surgical complication (transient, permanent, and hypothyroidism)	− 100% to + 100%	[14, 30]
**Costs**		
Price of [^18^F]FDG-PET/CT	€400–€5000	[14, 26, 27, 30, 87]
Price of HT	€2500–€20,000	[14, 26, 27, 30, 87]
Annual costs of observation after negative [^18^F]FDG-PET/CT	€0–€1000	[26, 30]
Costs of [^18^F]FDG-PET/CT incidental findings	€0–€1000	
**Disutility**		
Observation after negative [^18^F]FDG-PET/CT	0.00–0.10	[14, 26]
Observation after HT for benign nodule	0.00–0.10	[14, 26]

*EfFECTS*, observed data from the first year of the *EfFECTS* trial were included as a source. *HT*, hemithyroidectomy

## Results

Between July 2015 and October 2018, 132 adult patients with a Bethesda III or IV thyroid nodule were enrolled in the *EfFECTS* trial (Table 1). All patients completed all study-related procedures and 1-year follow-up. Diagnostic surgery was avoided for 25 of 91 (27%) patients in the [^18^F]FDG-PET/CT-driven group, as compared to 1 of 41 (2%) in the diagnostic surgery group (p = 0.001) [13]. The unoperated index nodules remained unchanged in size and unsuspicious on ultrasound surveillance and were considered benign after 1 year. During study follow-up, 106 (80%) patients underwent diagnostic surgery: 29 (22%) nodules were malignant, 8 (6%) were borderline tumours, and 69 (52%) were benign. For the [^18^F]FDG-PET/CT-driven group, this resulted in avoided futile diagnostic surgery for 25 of 62 (40%) benign nodules.

### First year utilities and costs

EQ-5D-5L, iMCQ and iPCQ questionnaires were fully completed at baseline, 3, 6, and 12 months by 121 (91.7%), 114 (86.4%), 107 (81.1%) and 106 (80.3%) of 132 patients, respectively, which were equally distributed across both randomisation groups. According to the EQ-5D-5L, the valuation of quality of life was similar in the [^18^F]FDG-PET/CT-driven and diagnostic surgery groups at all four measurements (Table 5). QALYs estimated from the EQ-5D-5L for the first year were similar in both groups (p = 0.57).

**Table 5:** Estimated utilities and quality adjusted life years (QALYs) per patient

	[^18^F]FDG-PET/CT- driven group	Diagnostic surgery group		
	(n = 91)	(n = 41)	Mean difference	*p*
**First year**				
Mean EQ-5D-5L domain scores:				
Baseline	0.852	0.791	0.061	0.14^a^
3 months	0.832	0.762	0.070	0.11^a^
6 months	0.749	0.674	0.075	0.23^a^
12 months	0.788	0.739	0.049	0.33^a^
Mean QALYs (95% CI)	0.778 (0.744–0.812)	0.759 (0.706–0.812)	0.019 (− 0.045– + 0.083)	0.57^b^
**Lifelong**				
Mean QALYs (95% CI)	19.273 (18.920–19.627)	18.871 (17.937–19.805)	0.402 (− 0.581– + 1.386)	0.42^b^

^a^ Unequal variances t-test. ^b^ generalized linear model. *QALYs*, quality-adjusted life years

The medical costs related to the index thyroid nodule were primarily determined by all regular healthcare consumption: a diagnostic workup, outpatient clinic visits, surgeries, medication, and RAI in case of malignancy (Table 6, Supplementary Table 4). In the [^18^F]FDG-PET/CT-driven group, additional costs were made for the [^18^F]FDG-PET/CT procedure (€754 per patient), but fewer diagnostic surgeries were performed, resulting in lower surgical costs per patient. Based on observed healthcare consumption, the mean costs for regular thyroid nodule-related healthcare were €6,100 in the [^18^F]FDG-PET/CT-driven group as compared to €7400 in the diagnostic surgery group, with a mean difference of –€1300 (p = 0.01). Additional healthcare consumption due to incidental findings on the [^18^F]FDG-PET/CT (e.g., costs for additional ultrasound and/or FNAC procedures for an [^18^F]FDG-positive thyroid incidentaloma) increased the medical costs in the [^18^F]FDG-PET/CT-driven group. This reduced the cost difference between both strategies to a mean difference of − €1,000 (p = 0.06) in thyroid nodule-related medical costs. Costs for surgical complications and other healthcare consumption (i.e., care unrelated to the thyroid nodule), patient costs, and productivity losses were similar across both groups. The total first-year societal costs were €15,500 in the [^18^F]FDG-PET/CT-driven group as compared to €20,100 in the diagnostic surgery group, with a mean difference of − €4500 (p = 0.06).

**Table 6:** Estimated 1-year and lifelong societal costs per patient

	[^18^F]FDG-PET/CT-driven group (*n* = 91)	Diagnostic surgery group (*n* = 41)		
	Mean costs per patient (95% CI)	Mean costs per patient (95% CI)	Mean difference (95% CI)	*p*^a^
***First year societal costs***				
**Medical costs**				
Thyroid nodule-related care				
Regular care	€6100 (€5400–€6800)	€7400 (€6600–€8100)	− €1300 (− €2,300– − €300)	**0.01**
Care related to [^18^F]FDG-PET incidental findings	€200 (€50–€350)	− €50 (− €100– + €0)	€200 (− €50– + €400)	**0.01**
Care related to surgical complications	€250 (€50–€400)	€200 (€0–€400)	€0 (− €250– + €250)	0.94
*Subtotal Thyroid nodule-related care*	*€6500 (€5700–€7300)*	*€7600 (€6800–€8300)*	− *€1000 (*− *€2100–€0)*	*0.06*
Other health care consumption	€2200 (€1500–€3000)	€3200 (€1100–€5200)	− €1000 (− €3000– + €1100)	0.36
*SUBTOTAL Medical costs*	*€8700 (€7600–€9800)*	*€10,700 (€8500–€13,000)*	− *€2000 (*− *€4400–* + *€400)*	*0.10*
**Patient costs**				
Travel expenses	€150 (€150–€200)	€200 (€100–€300)	− €50 (− €150– + €50)	0.31
Informal care	€450 (€100–€850)	€900 (€150–€1700)	− €450 (− €1300– + €350)	0.27
*SUBTOTAL Patient costs*	*€650 (€250–€1000)*	*€1100 (€350–€1900)*	− *€500 (*− *€1300–* + *€300)*	*0.23*
**Productivity losses**				
Paid productivity losses	€5200 (€3800–€6500)	€6800 (€4300–€9300)	− €1600 (− €4400– + €1200)	0.25
Unpaid productivity loss	€1000 (€600–€1400)	€1400 (€700–€2200)	− €400 (− €1200– + €450)	0.35
*SUBTOTAL Productivity losses*	*€6200 (€4750–€7650)*	*€8200 (€5500–€10,950)*	− *€2050 (*− *€5100–* + *€1050)*	*0.19*
TOTAL First year societal costs	€15,500 (€13,400–€17,700)	€20,100 (€15,800–€24,300)	− €4500 (− €9200– + €150)	0.06
***Lifelong societal costs***				
**Medical costs**				
Thyroid nodule-related care	€9100 (€7,900–€10,300)	€10,600 (€8800–€12,400)	− €1500 (− €3600– + €600)	0.17
Other health care consumption	€36,250 (− €121,200–€193,700)	€39,500 (− €119,800–€198,800)	− €3300 (− €8900– + €2300)	0.25
*SUBTOTAL Medical costs*	€45,300 (− €112,100– + €202,800)	€50,100 (− €109,200– + €209,400)	− €4800 (− 11,600– + €2,000)	0.17
**Patient costs**				
Travel expenses	€1900 (− €2700– + €6400)	€2000 (− €2600– + €6600)	− €150 (− €300– + €50)	0.11
Informal care	€10,800 (− €35,300– + €57,000)	€12,400 (− €34,000– + €58,800)	− €1500 (− €4000– + €1000)	0.23
*SUBTOTAL Patient costs*	*€12,700 (− €33,600–* + *€59,000)*	*€14,400 (*− *€32,200–* + *€60,900)*	− *€1700 (*− *€4300–* + *€900)*	*0.21*
**Productivity losses**				
Paid productivity losses	€27,200 (− €90,500– + €144,900)	€29,400 (− €89,700– + €148,500)	− €2200 (− €7400– + €2900)	0.40
Unpaid productivity loss	€18,200 (− €39,900– + €76,400)	€19,500 (− €39,300– + €78,300)	− €1200 (− €3300– + €800)	0.25
*SUBTOTAL Productivity losses*	*€45,500 (− €85,500–€176,400)*	*€48,900 (*− *€83,500–* + *€181,300)*	− *€3500 (*− *€9600–* + *€2600)*	*0.27*
TOTAL Lifelong societal costs	€103,500 (− €105,500– + €312,500)	€113,400 (− €98,200– + €325,000)	− €9900 (− €23,100– + €3200)	0.14

^a^ Generalized linear model. *95% CI*, 95% confidence interval

### Lifelong utilities and costs

Estimated using our Markov model, the lifelong utilities were similar for both strategies, with 19.273 mean QALYs for the [^18^F]FDG-PET/CT-driven group and 18.871 for the diagnostic surgery group (p = 0.42).

None of the lifelong societal costs were statistically significantly different between the two groups (Table 6). The mean discounted lifelong societal costs were €103,500 per patient in the [^18^F]FDG-PET/CT-driven group as compared to €113,400 in the diagnostic surgery group, with a mean difference of − €9,900 (p = 0.14). Lifelong extrapolation thus increased the size of the difference in QALYs and costs without reaching statistical significance.

### Cost-effectiveness analysis

**Fig. 3:**
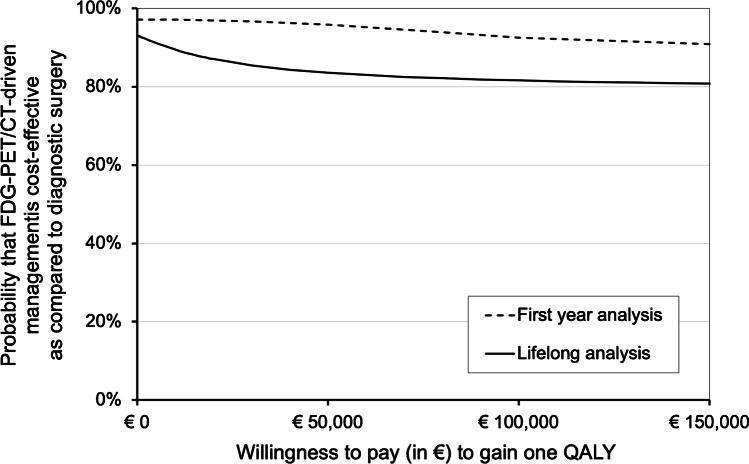
Cost-effectiveness acceptability curve (CEAC). For increasing willingness-to-pay thresholds, this figure shows the probability that [^18^F]FDG-PET/CT-driven management is cost-effective as compared to diagnostic surgery. Analysis was performed for the first-year (dashed line) and lifelong (continuous line) cost-effectiveness analysis

From a societal perspective, lifelong costs appeared in favour of [^18^F]FDG-PET/CT-driven management while HRQoL was sustained. Consequently, according to our analysis, [^18^F]FDG-PET/CT-driven management is very likely cost-effective as compared to diagnostic surgery for Bethesda III/IV thyroid nodules, regardless of the willingness to pay per QALY. The probability of cost-effectiveness is > 80% for any willingness to pay and minimally varies over the range of willingness to pay. The probability is 87% at €20,000 per QALY, 84% at €50,000, and 82% at €80,000 per QALY (Fig. 3).

### Univariate sensitivity analysis

**Fig. 4:**
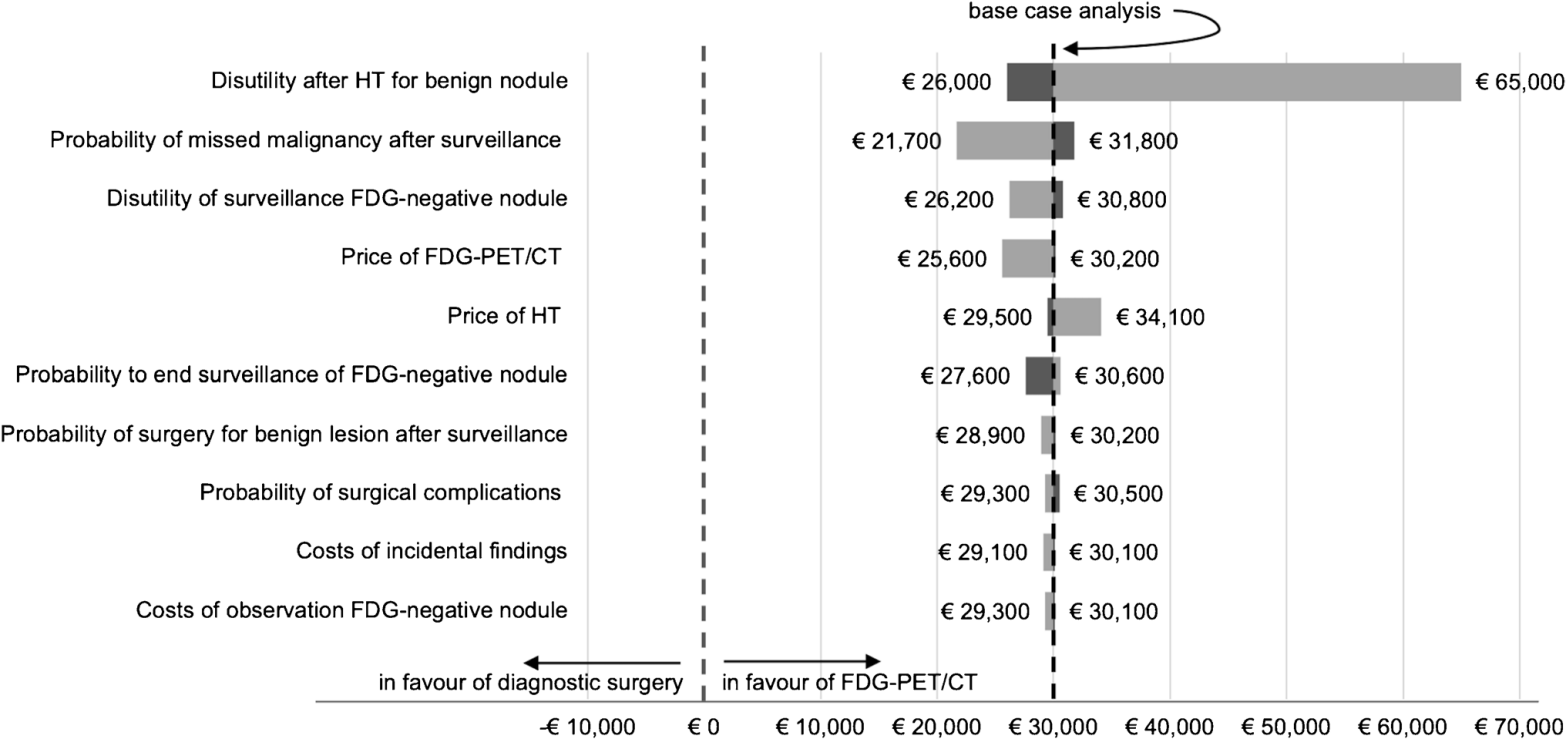
Tornado plot showing the results of the univariate sensitivity analysis on the lifelong incremental net monetary benefit per patient (x-axis) of [^18^F]FDG-PET/CT-driven management as compared to diagnostic surgery, for a willingness to pay of €50,000 per QALY. Dark grey bars represent lower parameter values and light grey bars represent higher parameter values. The vertical line at €0 represents the break-even situation, i.e., when both strategies have equal net benefit. The vertical line at €30,000 represents the incremental net benefit of the base case analysis for a willingness to pay of €50,000 per QALY. HT, hemithyroidectomy. QALY, quality-adjusted life year

Results of the univariate sensitivity analysis are shown in Fig. 4. At a willingness-to-pay of €50,000 per QALY, [^18^F]FDG-PET/CT-driven management remained cost-effective as compared to diagnostic surgery for the predetermined ranges of all of the parameters tested. Of the parameters selected for univariate sensitivity analysis, the disutility after HT for a benign nodule, the probability of a missed malignancy after initial surveillance for an [^18^F]FDG-negative nodule (representing the false-negative rate or NPV of [^18^F]FDG-PET/CT), the disutility of active surveillance of an [^18^F]FDG-negative nodule, and the price of the [^18^F]FDG-PET/CT had the largest influence on cost-effectiveness to the detriment of [^18^F]FDG-PET/CT-driven management.

## Discussion

The *EfFECTS* trial compared an [^18^F]FDG-PET/CT-driven diagnostic workup to diagnostic surgery in Bethesda III and IV thyroid nodules and previously demonstrated that [^18^F]FDG-PET/CT ensured an oncologically safe 40% reduction in diagnostic surgery for benign nodules, accurately ruling out malignancy with a sensitivity of 94.1% [13]. The current cost-utility study demonstrated that an [^18^F]FDG-PET/CT-driven workup reduced the 1-year thyroid nodule-related and societal costs. The clear 1-year cost differences persisted over the lifelong period, albeit with a larger 95% CI due to additional modelling uncertainties. Sustained HRQoL was observed over the first year as well as the lifelong period. Consequently, an [^18^F]FDG-PET/CT-driven is very likely cost-effective as compared to diagnostic surgery for Bethesda III/IV nodules.

The current study is in line with the results of the previous cost-effectiveness study from our group, which reported modelled cost-effectiveness of [^18^F]FDG-PET/CT-driven management in a Dutch setting over a 5-year horizon and provided the rationale for the *EfFECTS* trial [14]. According to that study, [^18^F]FDG-PET/CT was dominant over three reported alternative strategies, reducing costs while preserving HRQoL with an incremental net benefit of €3700, €1000, and €3900 as compared to diagnostic surgery or management driven by one of two commercial molecular marker panels, respectively. These two specific molecular marker panels have greatly evolved over the recent years and improved their diagnostic accuracy. It is likely that the cost-utility balance has changed in their favour. However, at $3600 per test (i.e., €3109; €1 = $1.13 on 10–01-2022, Medicare reimbursement rate [29]), nearing the costs of a hemithyroidectomy procedure, cost-effectiveness of these molecular marker panels likely remains challenging in a European setting.

Approximately a dozen cost-effectiveness studies are currently available on the use of commercially available molecular marker panels in indeterminate thyroid nodules. These mostly American studies generally focussed on the direct medical costs for thyroid-nodule related care only, including the costs and utilities for molecular testing, surgery, potential surgical complications, and (postoperative) observation. From different types of cost-effectiveness models, mixed conclusions regarding cost-effectiveness were reached [26, 29, 30, 35]. Nicholson et al. reported that both the Afirma® GSC and ThyroSeq® v3 were superior to diagnostic surgery [29]. In contrast, Balentine et al. demonstrated that diagnostic surgery was less costly and more effective than Afirma® GEC testing and ultrasound surveillance over a 5-year period. Similar to the results of the current study, their results proved sensitive to the estimated postoperative utilities and those of surveillance after a negative test [26]. Hu et al. recently demonstrated that selective molecular testing (i.e., molecular testing following a *repeat* Bethesda III or *single* Bethesda IV result) admittedly prevented 9.5% fewer diagnostic surgeries for benign nodules than reflexive molecular testing (i.e., molecular testing following *any first* Bethesda III or IV result), but was likely the cost-effective strategy due to the high costs of molecular testing. Their results were most sensitive to the costs of molecular testing [35].

The number of cost-effectiveness studies from a European perspective is limited. A recent study from a Dutch perspective estimated that molecular testing may save a considerable number of repeat FNAC procedures and diagnostic surgeries in Bethesda III and V nodules, resulting in a net saving of €100 and €4100 for these cytological categories, respectively. Unfortunately, the study excluded Bethesda IV nodules from their analysis [5].

To the best of our knowledge, the current study is the first cost-utility analysis on additional diagnostics in indeterminate thyroid nodules to be performed alongside a randomised controlled clinical trial. This contrasts our study with previous cost-utility analyses and provides a unique perspective. Our observed first-year healthcare consumption data and quality of life assessments are unparalleled, especially in patients with indeterminate thyroid nodules. By incorporating these data into a comprehensive lifelong cost-utility model, we presented a scenario that most accurately reflects real-world clinical practice. In contrast, most previous cost-utility studies used a theoretical base case, a more simplified model, somewhat idealized parameters, and/or a limited time horizon. Any lifelong HRQoL effects and (lifelong) costs other than the direct medical costs (i.e., costs for other health care consumption, patient costs, and productivity losses) were often disregarded in these studies [14, 26–28, 35].

In previous studies, the possibility of patient crossover between management strategies over time was also seldom taken into account [14, 26–28, 35]. We previously recognized that the therapeutic yield of [^18^F]FDG-PET/CT is influenced by patient preference and treatment compliance. This directly reflects on health-care consumption volumes and costs. Shared decision-making is crucial to carefully determine the most suitable management strategy for individual patients and prevent noncompliance, as well as to optimize the use of valuable diagnostic resources [13]. This is a dynamic process, in which preferences and interests may change as time passes. In studies on the natural course of cytologically benign nodules, up to 24% of nodules were surgically resected as time passed, primarily due to compressive symptoms [36–38]. It is important to acknowledge the dynamics of clinical practice in a cost-effectiveness model, too, as this may prevent overestimation of an effect of any given strategy. To account for this, our model included a yearly probability of surgery despite a negative [^18^F]FDG-PET/CT, a probability that surveillance of an [^18^F]FDG-negative nodule would end, and a probability to re-enter active surveillance after it had previously ended (Table 2, Fig. 2).

For the Markov model, we used triangular distributions for probabilities (Table 2) and utilities (Table 3) when uncertainty about these parameters was asymmetric. The base-case parameter value was the *mode* of the triangular distribution. Due to the asymmetry, the *mean* parameter value in the analysis was typically higher than the base-case value (by on average 18%, at most 67%). For the utilities, the higher mean of some parameters could be in favour of the [^18^F]FDG-PET/CT-driven group (e.g., utilities concerning surgery for benign disease and concurrent complications); others could be disadvantageous to the [^18^F]FDG-PET/CT-driven group (e.g., utilities concerning active surveillance of [^18^F]FDG-negative nodules). For the probabilities, the higher means were typically disadvantageous to the [^18^F]FDG-PET/CT-driven group (e.g., the probability of surgery for benign lesion after continued surveillance for [^18^F]FDG-negative nodules or the probabilities of complications due to thyroid surgery beyond the first year). Altogether, we believe the use of asymmetric triangular distributions was likely disadvantageous to the [^18^F]FDG-PET/CT-driven group and may have underestimated its cost-effectiveness, which was nevertheless more favourable than in the diagnostic surgery group.

As a Markov model remains a simplified reflection of the real situation, this is a limitation of any model-based cost-utility analysis and thus also applicable to the current study. The accuracy of the estimated probabilities, costs, and utilities are dependent on the availability and quality of representative source data. Although we performed a comprehensive literature search to ensure a careful, evidence-based determination of all model parameters, the best fitting literature for some variables was only moderately related. In these cases, an expert panel was additionally consulted. This included all parameters concerning the active surveillance of [^18^F]FDG-negative indeterminate thyroid nodules, for which we had to rely on literature about benign nodules and expert opinion. For example, a disutility of 0.02 was assigned to the active surveillance health state. We chose a limited but conservative disutility as compared to the disutility of observation after an uncomplicated HT for a benign nodule (0.01) to prevent overestimation of HRQoL in favour of an [^18^F]FDG-PET/CT-driven workup and to account for any suspense of not knowing a definite histopathological diagnosis. Patients under surveillance may experience some degree of cyclic psychological distress centering around their yearly follow-up visits, although evidence supporting that assumption is currently lacking and we have not observed it in the *EfFECTS* trial [26]. A recent study with a limited median 15-month follow-up found no evidence of such effects and showed sustained HRQoL in patients under surveillance following a negative molecular test [39]. We included the disutilities of both observation after HT and observation after a negative [^18^F]FDG-PET/CT scan in our univariate sensitivity analysis. Although these disutilities did affect the incremental net benefit, [^18^F]FDG-PET/CT remained the cost-effective strategy across the tested ranges.

In conclusion, the current cost-utility study showed that an [^18^F]FDG-PET/CT-driven diagnostic workup reduced the 1-year thyroid nodule-related and societal costs while sustaining quality of life. Following the observed reduction in diagnostic surgery for benign nodules, an [^18^F]FDG-PET/CT-driven workup is very likely cost-effective from a Dutch societal perspective as compared to diagnostic surgery for Bethesda III/IV nodules.

## Supplementary Information

Below is the link to the electronic supplementary material.

Supplementary file1 (DOCX 109 KB)

## Data Availability

The study protocol and datasets generated during and/or analysed during the current study are available from the corresponding author on reasonable request. Data requestors will need to sign a data access agreement and in keeping with patient consent for secondary use, obtain ethical approval for any new analyses.
