# Supplementary material for: FDG-PET/CT in indeterminate thyroid nodules: cost-utility analysis alongside a randomised controlled trial
Source: Eur J Nucl Med Mol Imaging. 2022 Apr 18;49(10):3452–69. doi: 10.1007/s00259-022-05794-w (PMC9308600; doi:10.1007/s00259-022-05794-w)
Supplement: Supplementary file 1 — Supplementary file1 (DOCX 109 KB) [file 259_2022_5794_MOESM1_ESM.docx]

Supplementary Data

**FDG-PET/CT in indeterminate thyroid nodules:**

**cost-utility analysis alongside a randomised controlled trial**

Elizabeth J. de Koster, Dennis Vriens, Maarten O. van Aken, Lioe-Ting Dijkhorst-Oei,

Wim J.G. Oyen, Robin P. Peeters, Abbey Schepers, Lioe-Fee de Geus-Oei, Wilbert B. van den Hout

for the *EfFECTS* trial study group.

**Table of Contents**

**EfFECTS trial study group 3.**

Trial steering committee 3.

Local principal investigators 3.

Study safety committee 4.

**Supplementary Data 5.**

Supplementary Table 1: Markov Model: Linear regression analysis to establish 5.

estimated costs for non-thyroid-related costs

Supplementary Table 2: Markov Model: Yearly travel expenses, thyroid-related, 6.

per health state

Supplementary Table 3: Unadjusted estimated utilities and quality adjusted life 7.

years (QALYs).

Supplementary Table 4. Unadjusted estimated mean societal costs during the first year. 8.

Supplementary Table 5. Unadjusted estimated lifelong mean societal costs. 9.

Supplementary Figure 1. Cost-effectiveness acceptability curve (CEAC), unadjusted 10.

analysis.

**EfFECTS trial study group**

**Trail steering committee**

- Prof. dr. L.F. de Geus-Oei, MD PhD (**project leader**), *Leiden University Medical Center, Department of Radiology, Section of Nuclear Medicine, Leiden, the Netherlands; Radboud University Medical Centre, Department of Radiology and Nuclear Medicine, Nijmegen, the Netherlands*
- Prof. dr. W.J.G. Oyen, MD PhD (**principal investigator**), *Radboud University Medical Centre, Department of Radiology and Nuclear Medicine, Nijmegen, the Netherlands; Rijnstate Hospital, Department of Radiology and Nuclear Medicine, Arnhem, the Netherlands; Department of Biomedical Sciences and Humanitas Clinical and Research Centre, Department of Nuclear Medicine, Humanitas University, Milan, Italy*
- Dr. D. Vriens, MD PhD (**principal investigator**), *Leiden University Medical Center, Department of Radiology, Section of Nuclear Medicine, Leiden, the Netherland.*
- E.J. de Koster, MD (**junior investigator**), *Radboud University Medical Centre, Department of Radiology and Nuclear Medicine, Nijmegen, the Netherlands*

**Local principal investigators**

**Radboud university medical centre, Nijmegen, the Netherlands**

- Dr. A.C.H. van Engen-van Grunsven, MD PhD, *Department of Pathology*
- E.J. de Koster, MD, *Department of Radiology and Nuclear Medicine*
- Dr. B. Küsters, MD PhD, *Department of Pathology*
- Prof. dr. R.T. Netea-Maier, MD PhD, *Department of Internal Medicine, Division of Endocrinology*
- Prof. dr. J.W.A. Smit, MD PhD, *Department of Internal Medicine, Division of Endocrinology*
- Prof. dr. J.H.W. de Wilt, MD PhD, *Department of Surgical Oncology*

**Amsterdam University Medical Centers, Amsterdam, the Netherlands**

*Location Academic Medical Center*

- Prof. dr. J. Booij, MD PhD, *Department of Radiology and Nuclear Medicine*
- Prof. dr. E. Fliers, MD PhD, *Department of Endocrinology and Metabolism*
- Dr. T.K. Klooker, MD PhD, *Department of Endocrinology and Metabolism*

*Location VU University Medical Center*

- Dr. E.W.C.M. van Dam, MD PhD, *Department of Internal Medicine, Division of Endocrinology*
- Dr. K.M.A. Dreijerink, MD PhD, *Department of Internal Medicine, Division of Endocrinology*
- Dr. P.G.H.M. Raijmakers, MD PhD, *Department of Radiology and Nuclear Medicine*

**Erasmus University Medical Centre, Rotterdam, the Netherlands**

- Dr. B.L.R. Kam, MD PhD, *Department of Nuclear Medicine*
- Prof. dr. R.P. Peeters, MD PhD, *Department of Internal Medicine*
- Prof. dr. J. Verzijlbergen, MD PhD, *Department of Nuclear Medicine*

**Haga Hospital, The Hague, the Netherlands**

- Dr. M.O. van Aken, MD PhD, *Department of Internal Medicine*

**Isala Hospital, Zwolle, the Netherlands**

- Prof. dr. P.L. Jager, MD PhD, *Department of Nuclear Medicine*
- Dr. G.S. Mijnhout, MD PhD, *Department of Internal Medicine*

**Leiden University Medical Center, Leiden, the Netherlands**

- Prof. dr. L.F. de Geus-Oei, MD PhD, *Department of Radiology, Section of Nuclear Medicine*
- Dr. W.B. van den Hout, PhD, *Department of Biomedical Data Sciences-Medical Decision Making*
- Prof. dr. A.M. Pereira Arias, MD PhD, *Department of Internal Medicine, Division of Endocrinology*
- Prof. dr. J. Morreau, MD PhD, *Department of Pathology*
- Dr. M. Snel, MD PhD, *Department of Internal Medicine, Division of Endocrinology*
- Dr. D. Vriens, MD PhD, *Department of Radiology, Section of Nuclear Medicine*

**Meander Medical Centre, Amersfoort, the Netherlands**

- Dr. L.T. Dijkhorst-Oei, MD PhD, *Department of Internal Medicine*
- Dr. J.M.H. de Klerk, MD PhD, *Department of Nuclear Medicine*

**Maastricht University Medical Centre, Maastricht, the Netherlands**

- Dr. B. Havekes, MD PhD, *Department of Internal Medicine, Division of Endocrinology*
- Dr. D.C. Mitea, MD PhD, *Department of Radiology and Nuclear Medicine*
- Dr. S. Vöö, MD PhD, *Department of Radiology and Nuclear Medicine*

**OLVG Hospital, Amsterdam, the Netherlands**

- Dr. C.B. Brouwer, MD PhD, *Department of Internal Medicine*
- Dr. P.S. van Dam, MD PhD, *Department of Internal Medicine*
- Dr. F. Sivro, MD PhD, *Department of Nuclear Medicine*

**Reinier de Graaf Hospital, Delft, the Netherlands**

- Dr. E.T. te Beek, MD PhD, *Department of Nuclear Medicine*
- Dr. M.C.W. Jebbink, MD PhD, *Department of Internal Medicine*

**Rijnstate Hospital, Arnhem, the Netherlands**

- Dr. G.S. Bleumink, MD PhD, *Department of Internal Medicine*
- Prof. dr. W.J.G. Oyen, MD PhD, Department of Radiology and Nuclear Medicine
- Dr. V.J.R. Schelfhout, MD PhD, Department of Radiology and Nuclear Medicine

**St. Antonius Hospital, Nieuwegein, the Netherlands**

- Dr. R.G.M. Keijsers, MD PhD, *Department of Nuclear Medicine*
- Dr. I.M.M.J. Wakelkamp, MD PhD, *Department of Internal Medicine*

**University Medical Centre Groningen, Groningen, the Netherlands**

- Dr. A.H. Brouwers, MD PhD, *Department of Nuclear Medicine and Molecular Imaging*
- Prof. dr. T.P. Links, MD PhD, *Division of Endocrinology, Department of Internal Medicine*

**University Medical Centre Utrecht, Utrecht, the Netherlands**

- Dr. B. de Keizer, MD PhD, *Department of Radiology and Nuclear Medicine*
- Dr. R. van Leeuwaarde, MD PhD, *Department of Endocrine Oncology*

**Study safety committee**

- Dr. J.J. Bonenkamp, MD PhD, *Department of Surgical Oncology, Radboud University Medical Centre, Nijmegen, The Netherlands.*
- Dr. A.R.T. Donders, PhD, *Department for Health Evidence, Radboud University Medical Centre, Nijmegen, the Netherlands*
- Prof. dr. J.J. Fütterer, Phd, *Department of Radiology and Nuclear Medicine, Radboud University Medical Centre, Nijmegen, The Netherlands.*

**Supplementary Table 1.**

**Markov model: Linear regression analysis to establish estimated costs for non-thyroid-related costs**

|  | **Mean costs (€)** | **Standard error** | **Source** |
| --- | --- | --- | --- |
| **Non-Thyroid related health care** | | | |
| **Mean yearly other health care consumption** | € 2,511.26 (mean) |  | *EfFECTS* |
| Regression (Constant) | € 10,004.52 | € 3,620.51 |  |
| QALYs | -€ 7,191.73 | € 2,695.88 |  |
| Age at baseline (years) | € 15.00 | € 35.48 |  |
| Sex (0=male / 1=female) | -€ 3,397.72 | € 1,253.27 |  |
| **Yearly travel expenses for other health care consumption, non-thyroid related** | € 98.83 (mean) |  | *EfFECTS* |
| Regression (Constant) | € 164.37 | € 105.30 |  |
| QALYs | -€ 143.68 | € 87.08 |  |
| Age at baseline (years) | € 1.04 | € 1.05 |  |
| Sex (0=male / 1=female) | -€ 13.64 | € 36.25 |  |
| **Informal care** | € 603.55 (mean) |  | *EfFECTS* |
| Regression (Constant) | € 5,111.33 | € 1,086.99 |  |
| QALYs | -€ 5,437.58 | € 1,322.19 |  |
| Age at baseline (years) | € 0 |  |  |
| Sex (0=male / 1=female) | € 0 |  |  |
| **Productivity losses** | | | |
| **Yearly other paid productivity losses** | € 2,267.41 (mean) |  | *EfFECTS* |
| Regression (Constant) | € 7,211.94 | € 2,709.56 |  |
| QALYs | -€ 1,429.17 | € 2,153.16 |  |
| Age at baseline (years) | -€ 64.69 | € 29.51 |  |
| Sex (0=male / 1=female) | -€ 396.87 | € 1,049.30 |  |
| **Yearly unpaid productivity losses** | € 1,152.85 (mean) |  | *EfFECTS* |
| Regression (Constant) | € 3,153.51 | € 1,337.83 |  |
| QALYs | -€ 3,487.51 | € 1,110.21 |  |
| Age at baseline (years) | € 5.95 | € 14.29 |  |
| Sex (0=male / 1=female) | € 455.50 | € 502.93 |  |

*EfFECTS*, observed data from the first year of the *EfFECTS* trial were included as a source. QALYs, quality adjusted life years

**Supplementary Table 2.**

**Markov model: yearly travel expenses, thyroid-related, per health state**

|  | **Costs, Expected value** | **Reference** | **Uncertainty range for PSA^a^** |
| --- | --- | --- | --- |
| **Yearly travel expenses, thyroid related** | | | |
| **Any procedure, onetime costs (i.e., HT, (c)TT, radioiodine ablation)** | €58 | *EfFECTS* | ±25% |
| **Observation after negative FDG-PET** | €17 | *EfFECTS* | ±25% |
| **End of follow-up** | €0 | *EfFECTS* |  |
| **Observation after HT for benign nodule** |  | *EfFECTS* |  |
| 1^st^ year | €23 |  | ±25% |
| 2^nd^ year onwards | €0 |  |  |
| **Observation after HT for malignancy** |  | *EfFECTS* |  |
| 1^st^ year | €46 |  | ±25% |
| 2^nd^-5^th^ year | €23 |  | ±25% |
| 6^th^ year onwards | €0 |  |  |
| **Transient complication due to HT** | €12 | *EfFECTS* | ±25% |
| **Permanent complication due to HT** | €23 | *EfFECTS* | ±25% |
| **Hypothyroidism due to HT** |  | *EfFECTS* |  |
| 1^st^ year | €46 |  | ±25% |
| 2^nd^ year onwards | €23 |  | ±25% |
| **Recurrence of malignancy after HT** | €70 | *EfFECTS* | ±25% |
| **Observation after TT for benign nodule** |  | *EfFECTS* |  |
| 1^st^ year | €70 |  | ±25% |
| 2^nd^ year onwards | €23 |  | ±25% |
| **Observation after (c)TT for malignancy** |  | *EfFECTS* |  |
| 1^st^ year | €104 |  | ±25% |
| 2^nd^-15^th^ year | €35 |  | ±25% |
| 16^th^ year onwards | €0 |  | 0 |
| **Transient complication due to (c)TT** | €12 | *EfFECTS* | ±25% |
| **Permanent complication due to (c)TT** | €23 | *EfFECTS* | ±25% |
| **Recurrence after (c)TT** | €70 | *EfFECTS* | ±25% |
| **Death** | €0 | *EfFECTS* |  |

^a^: a triangular distribution was used for the uncertainty ranges of the probabilistic sensitivity analyses. *EfFECTS*, observed data from the first year of the *EfFECTS* trial were included as a source.

**Supplementary Table 3.**

**Unadjusted estimated utilities and quality adjusted life years (QALYs) per patient.**

|  | **[^18^F]FDG-PET/CT- driven group** | **Diagnostic surgery group** |  |  |
| --- | --- | --- | --- | --- |
|  | **(n=91)** | **(n=41)** | **mean difference** | ***p*** |
| **First year** | | | | |
| Mean QALYs (95% CI) | 0.792 (0.749-0.836) | 0.727 (0.663-0.791) | 0.065 (-0.018-+0.148) | 0.12^a^ |
| **Second year onwards** |  |  |  |  |
| Mean QALYs (95% CI) | 18.389 (17.017-19.760) | 18.350 (16.315-20.385) | 0.035 (-2.094-+2.165) | 0.97^a^ |
| **Lifelong QALYs** |  |  |  |  |
| Mean QALYs (95% CI) | 19.181 (17.809-20.552) | 19.077 (17.042-21.112) | 0.104 (-2.033-+2.240) | 0.92^a^ |

^a^: unequal variances t-test. QALYs, quality-adjusted life years.

**Supplementary Table 4.**

**Unadjusted estimated 1-year mean societal costs per patient.**

|  |  | | |  | **Observed data, unadjusted analysis** | | | | | |
| --- | --- | --- | --- | --- | --- | --- | --- | --- | --- | --- |
|  |  | | |  | **[^18^F]FDG-PET/CT-driven group (n=91)** | | **diagnostic surgery**  **(n=41)** | |  |  |
| **FIRST YEAR SOCIETAL COSTS** | | | | | mean volume per patient | mean costs per patient  (95% CI) | mean volume per patient | mean costs per patient  (95% CI) | mean difference  (95% CI) | *p*^a^ |
| **Medical costs: thyroid nodule-related care** | | | | |  |  |  |  |  |  |
| *Regular care* | | | | |  |  |  |  |  |  |
|  | Surgical costs | | | |  |  |  |  |  |  |
|  |  | | | Diagnostic surgery | 66^c^ | €2,601 | 40^c^ | €3,366 | -€765 | **<0.001** |
|  |  | | | Hospital admission days for diagnostic surgery | 1.5 (0-3)^d^ | €630 | 2.1 (0-3)^d^ | €886 | -€256 | **<0.001** |
|  |  | | | Completion thyroidectomy | 13^c^ | €750 | 4^c^ | €512 | €238 | 0.45 |
|  |  | | | Hospital admission days for cTT | 0.3 (0-4)^d^ | €128 | 0.2 (0-4)^d^ | €105 | €23 | 0.72 |
|  | Radioiodine therapy | | | | 12^b^ | €803 | 4^c^ | €621 | €182 | 0.63 |
|  | Outpatient clinic visits | | | | 6.0 (2-23)^d^ | €714 | 5.6 (3-11)^d^ | €660 | €54 | 0.40 |
|  | Day treatment | | | | 0.0 (0-4)^d^ | €47 | 0^d^ | €0 | €47 | 0.32 |
|  | Emergency room | | | | 0.0 (0-2)^d^ | €9 | 0^d^ | €0 | €9 | 0.18 |
|  | [^18^F]FDG-PET/CT | | | | 1 (1)^d^ | €754 | 0^d^ | €0 | €754 | **<0.001** |
|  | Blood tests (e.g., thyroid function) | | | | 2.1 (0-11)^d^ | €66 | 2.2 (0-5)^d^ | €64 | €2 | 0.83 |
|  | Other diagnostics (e.g., imaging, FNAC) | | | | 0.6 (0-3)^d^ | €68 | 0.3 (0-4)^d^ | €34 | €33 | 0.11 |
|  | Medication | | | | 29 (32%)^c^ | €23 | 13 (32%)^c^ | €12 | €11 | 0.32 |
|  | *Subtotal* | | | |  | *€6,592* |  | *€6,260* | *€332*  *(-€1,428-+€2,092)* | *0.71* |
|  |  |  |  |  | *(€5,489-€7,696)* | | *(€4,617-€7,904)* | |  |  |
| *Care related to [^18^F]FDG-PET incidental findings* | | | | | 11^c^ |  | n.a. |  |  |  |
|  | Surgical costs | | | | 4^c^ | €79 |  | €0 | € 79 | 0.04 |
|  | Hospital admission days | | | | 0.0 (0-1)^d^ | €2 |  | €0 | € 2 | 0.32 |
|  | Outpatient clinic visits | | | | 0.4 (0-10)^d^ | €47 |  | €0 | € 47 | **0.01** |
|  | Blood tests (e.g., thyroid function) | | | | 0.2 (0-5)^d^ | €4 |  | €0 | € 4 | **0.05** |
|  | Other diagnostics (e.g., imaging, FNAC) | | | | 0.2 (0-4)^d^ | €44 |  | €0 | € 44 | **0.00** |
|  | Medication | | | | 4 (4%)^d^ | €3 |  | €0 | € 3 | 0.06 |
|  | *Subtotal* | | | |  | *€180* |  | *€0* | *€180*  *(-€40-+€319)* | ***0.01*** |
|  |  |  |  |  |  | *(€64-€295)* |  | *(-€172-+€172)* |  |  |
| *Care related to surgical complications* | | | | | 19^c^ |  | 10^c^ |  |  |  |
|  | Surgical costs | | | | 3^c^ | €114 | 1^c^ | €84 | €30 | 0.78 |
|  | Hospital admission days | | | | 0.1 (0-4)^d^ | €52 | 0.0 (0-1)^d^ | €11 | €42 | 0.16 |
|  | Outpatient clinic visits | | | | 0.4 (0-6)^d^ | €43 | 0.6 (0-5)^d^ | €67 | -€25 | 0.38 |
|  | Emergency room | | | | 0.1 (0-2)^d^ | €18 | 0.0 (0-1)^d^ | €7 | €11 | 0.32 |
|  | Blood tests | | | | 0.2 (0-5)^d^ | €5 | 0.4 (0-4)^d^ | €9 | -€4 | 0.37 |
|  | Medication | | | | 10 (11%)^c^ | €7 | 9 (22%)^c^ | €15 | -€8 | 0.20 |
|  | *Subtotal* | | | |  | *€239* |  | *€193* | *€46*  *(-€225-+€318)* | *0.74* |
|  |  |  |  |  |  | *(€71-€406)* |  | *(-€57-+€442)* |  |  |
| *Total medical costs: thyroid nodule-related care* | | | | |  | *€7,011* |  | *€6,453* | *€558*  *(-€1,232-+€2,347)* | *0.54* |
|  |  |  |  |  | *(€5,875-€8,147)* | | *(€4,761-€8,146)* | |  |  |
| **Medical costs: other health care consumption** | | | | |  |  |  |  |  |  |
|  | | | General practitioner | | 4.1^d^ | €146 | 5.1^d^ | €179 | -€33 | 0.32 |
|  | | | Social worker | | 0.1^d^ | €8 | 0.2^d^ | €13 | -€5 | 0.66 |
|  | | | Physiotherapist | | 4.5^d^ | €159 | 7.6^d^ | €268 | -€110 | 0.17 |
|  | | | Occupational therapist | | 0.1^d^ | € 2 | 0^d^ | €0 | €1 | 0.28 |
|  | | | Speech therapist | | 0.6^d^ | €23 | 1.6^d^ | €57 | -€35 | 0.53 |
|  | | | Dietician | | 0.5^d^ | €16 | 0.7^d^ | €20 | -€4 | 0.69 |
|  | | | Homeopath | | 1.1^d^ | €79 | 0.7^d^ | €54 | €25 | 0.54 |
|  | | | Psychologist or psychiatrist | | 1.9^d^ | €189 | 1.2^d^ | €116 | €73 | 0.28 |
|  | | | Occupational physician | | 0.5^d^ | €81 | 0.5^d^ | €79 | €2 | 0.97 |
|  | | | Emergency room | | 0.2^d^ | €43 | 0.2^d^ | €41 | €2 | 0.94 |
|  | | | Ambulance | | 0.3^d^ | €144 | 0.2^d^ | €112 | €32 | 0.81 |
|  | | | Outpatient clinic | | 2.0^d^ | €245 | 2.7^d^ | €326 | -€81 | 0.36 |
|  | | | In-hospital day treatment | | 0.3^d^ | €197 | 2.6^d^ | €707 | -€510 | 0.23 |
|  | | | Hospital admission days | | 0.3^d^ | €146 | 1.9^d^ | €941 | -€795 | 0.31 |
|  | | | Home care | | 4.0^e^ | €127 | 5.7^e^ | €125 | €2 | 0.99 |
|  | | | Medication | | 72 (79%)^c^ | €433 | 33 (80%)^c^ | €529 | -€96 | 0.75 |
| *Total medical costs: other health care consumption* | | | | |  | *€2,035* |  | *€3,568* | *-€1,534*  *(-€4,118-+€1,051)* | *0.25* |
|  |  |  |  |  | *(€936-€3,134)* | | *(€1,907-€5,230)* | |  |  |
| TOTAL Medical costs | | | | |  | €9,046 |  | €10,021 | -€976  (-€4,089-+€2,137) | 0.54 |
|  |  |  |  |  | (€7,470-€10,621) | | (€7,658-€12,385) | |  |  |
| **Patient costs** | | | | |  |  |  |  |  |  |
| Travel expenses | | | | |  |  |  |  |  |  |
|  | | Thyroid nodule related care | | | 7.9 (2-25)^f^ | €82 | 6.4 (3-14)^f^ | €63 | €19 | 0.04 |
|  | | Other health care | | | 16.0^f^ | €80 | 23.2^f^ | €140 | -€59 | 0.16 |
| *Total travel expenses* | | | | |  | *€162* |  | *€202* |  |  |
| Informal care | | | | | 16.3^e^ | €243 | 93.9^e^ | €1,404 | -€1,161 | 0.16 |
| TOTAL Patient costs | | | | |  | €405 |  | €1,606 | -€1,201  (-€2,820-+€418) | 0.15 |
|  |  |  |  |  | (-€225-+€1,035) | | (€667-€2,545) | |  |  |

**Supplementary Table 4 (*continued)*.**

|  |  |  | **Observed data, unadjusted analysis** | | | | | |
| --- | --- | --- | --- | --- | --- | --- | --- | --- |
|  |  |  | **[^18^F]FDG-PET/CT-driven group (n=91)** | | **diagnostic surgery group (n=41)** | |  |  |
| **FIRST YEAR SOCIETAL COSTS (*continued)*** | | | mean volume per patient | mean costs per patient  (95% CI) | mean volume per patient | mean costs per patient  (95% CI) | mean difference  (95% CI) | *p*^a^ |
| **Productivity losses** | | |  |  |  |  |  |  |
|  | Paid productivity following HT | | 69.7^e^ | €2,587 | 75.2^e^ | €2,791 | -€204 | 0.84 |
|  | Paid productivity loss following cTT | | 15.5^e^ | €574 | 18.0^e^ | €669 | -€96 | 0.84 |
|  | Paid productivity loss following RAI | | 5.1^e^ | €189 | 1.2^e^ | €43 | €146 | **0.05** |
|  | Other paid productivity loss | | 48.1^e^ | €1,785 | 90.0^e^ | €3,337 | -€1,552 | 0.13 |
| *Total paid productivity losses* | | |  | *€5,136* |  | *€6,841* | *-€1,705* | *0.32* |
| Unpaid productivity loss | | | 63.2^e^ | €944 | 108.1^e^ | €1,616 | -€671 | 0.20 |
| TOTAL Productivity losses | | |  | €6,080 |  | €8,457 | -€2,376  (-€6,029-+€1,276) | 0.20 |
|  |  |  | (€4,244-€7,917) | | (€5,535-€11,378) | |  |  |
| TOTAL Societal costs first year | | |  | €15,531 |  | €20,084 | -€4,553  (-€10,454-+€1,347) | 0.13 |
|  |  |  | (€12,572-€18,490) | | (€15,494-€24,674) | |  |  |

^a:^ unequal variances t-test. ^c^: total number of patients or procedures (%). ^d^: mean number of appointments/days per patient (range, if available). ^e^: mean number of hours per year per patient. ^f^: mean number of visits per patient (return trips, range). CI, confidence interval. cTT, completing total thyroidectomy. FNAC, Fine needle aspiration cytology. HT, hemithyroidectomy (including isthmus resection and nodulectomy). N.a., not applicable. RAI, radioiodine ablative therapy.

**Supplementary Table 5.**

**Unadjusted estimated lifelong mean societal costs per patient.**

|  | **[^18^F]FDG-PET/CT-driven group (n=91)** | **Diagnostic surgery group**  **(n=41)** |  |  |
| --- | --- | --- | --- | --- |
|  | mean costs per patient (95% CI) | mean costs per patient (95% CI) | mean difference (95% CI) | *p*^a^ |
| **Medical costs** |  |  |  |  |
| Thyroid nodule-related care | € 9,973 (€8,071-€11,876) | € 8,665 (€5,888-€11,442) | € 1,308 (-€1,954-+€4,571) | 0.43 |
| Other health care consumption | € 36,677 (-€120,417-+€193,771) | € 38,523 (-€121,981-+€199,027) | -€ 1,846 (-€13,834-+€10,147) | 0.76 |
| *SUBTOTAL Medical costs* | *€ 46,650 (-€110,486-+€203,787)* | *€ 47,188 (-€113,387-+€207,762)* | *-€ 537 (-€13,935-+€12,861)* | *0.94* |
| **Patient costs** |  |  |  |  |
| Travel expenses | € 1,885 (-€2,620-+€6,390) | € 1,955 (-€2,645-+€6,555) | -€ 70 (-€351-+€211) | 0.63 |
| Informal care | € 10,945 (-€35,106-+€56,996) | € 12,146 (-€34,644-+€58,937) | -€ 1,202 (-€4,552-+€1,709) | 0.48 |
| *SUBTOTAL Patient costs* | *€ 12,830 (-€33,354-+€59,013)* | *€ 14,101 (-€32,828-+€61,031)* | *-€ 1,272 (-€4,708-+€2,164)* | *0.47* |
| **Productivity losses** |  |  |  |  |
| Paid productivity losses | € 27,670 (-€89,758-€145,098) | € 28,398 (-€91,653-+€148,450) | -€ 728 (-€10,728-+€9,272) | 0.89 |
| Unpaid productivity loss | € 18,308 (-€39,709-+€76,325) | € 19,347 (-€39,929-+€78,622) | -€ 1,039 (-€4,662-+€2,585) | 0.57 |
| *SUBTOTAL Productivity losses* | *€ 45,978 (-€84,667-+€176,623)* | *€ 47,745 (-€85,759-+€181,249)* | *(-€13,144-+€9,610)* | *0.76* |
| **TOTAL Lifelong societal costs** | **€ 105,458 (-€103,180-€314,096)** | **€ 109,034 (-€104,189-€322,257)** | **-€ 3,576 (-€25,387-€18,235)** | **0.75** |

^a:^ unequal variances t-test.

**Supplementary Figure 1.**

Cost-effectiveness acceptability curve (CEAC), unadjusted analysis.
